# An AI-Driven Clinical Decision Support Model Based on Anemia and Fibroid Parameters to Guide Surgical Decision-Making

**DOI:** 10.3390/medicina62030555

**Published:** 2026-03-17

**Authors:** İnci Öz, Ecem Esma Yegin, Ali Utku Öz, Engin Ulukaya

**Affiliations:** 1 Department of Gynaecology of Obstetrics, Medicana Atakoy Hospital, 34158 Istanbul, Türkiye; 2 Department of Gynaecology of Obstetrics, Faculty of Medicine, Istinye University, 34010 Istanbul, Türkiye; 3 Molecular Cancer Research Center, Istinye University, 34396 Istanbul, Türkiyeeulukaya@istinye.edu.tr (E.U.); 4 Department of Biostatistics and Medical Informatics, Faculty of Medicine, Istinye University, 34010 Istanbul, Türkiye; 5 Department of Gynaecology of Obstetrics, Cam & Sakura City Hospital, 34480 Istanbul, Türkiye; 6 Department of Biochemistry, Faculty of Medicine, Istinye University, 34010 Istanbul, Türkiye

**Keywords:** surgery of uterine fibroid, artificial intelligence, machine learning, anemia, decision support algorithm

## Abstract

*Background and Objectives*: This study aimed to identify the clinical factors associated with the need for surgical intervention in women with uterine fibroids (UFs) and develop a data-driven clinical decision helper algorithm. By comparing hematologic and fibroid characteristics and prospectively assessing clinical concordance with the model predictions, we sought to create an objective tool for surgical decision-making. *Materials and Methods*: This retrospective study enrolled 618 women with UFs who were evaluated at three participating hospitals. Of these, 238 (38.5%) underwent surgery. Comparative statistical analyses were conducted between patients who underwent myomectomy and those who did not. Machine learning (ML) models were trained to predict myomectomy necessity. A clinical concordance assessment was conducted using 50 cases that were evaluated in real time by a gynecologist blinded to both the clinical outcomes and the model outputs. Agreement between clinical assessment and algorithm-based predictions was subsequently evaluated. *Results*: Hemoglobin and ferritin concentrations were significantly reduced in the surgery group compared with the non-surgery group (*p* < 0.001). ML analyses integrating fibroid characteristics with anemia-related markers identified support vector ML models as the most accurate classifiers. Ferritin-based models achieved accuracies of 98–99% and near-perfect ROC–AUC values. ML models combining UF number or volume with ferritin demonstrated the highest precision, sensitivity, and F1-scores. Clinical concordance analysis showed 98% agreement with the blinded gynecologist, with only one borderline discordant case. *Conclusions*: This decision helper algorithm provides a highly accurate and objective tool for predicting surgical necessity in patients with UFs. Anemia status and fibroid characteristics were the strongest predictors. By reducing subjective variability and closely reflecting expert reasoning, the model offers a practical framework for integration into routine gynecologic decision-making.

## 1. Introduction

Uterine fibroids (UFs) are benign neoplasms arising from the smooth muscle of the myometrium and are the most commonly encountered tumors in women worldwide [1]. Their prevalence varies considerably across studies—ranging from 5.4% to 77%—depending on population characteristics and diagnostic methods [2,3]

Management involves diverse medical therapies, with surgical intervention considered when clinically warranted [4,5]. In routine practice, the decision to proceed with surgery is shaped by a combination of symptom severity, hematologic status, fibroid burden, patient characteristics, and clinician experience. However, considerable inter-clinician variability exists, particularly among less-experienced providers.

Published clinical studies further emphasize that anemia and fibroid burden are among the strongest determinants of surgical management in UF patients. In a large retrospective cohort of 36,295 hospitalized women with leiomyomas, anemia was associated with substantially higher odds of requiring operative treatment and increased perioperative morbidity (OR 2.56 and OR 5.29, respectively), highlighting its critical role in guiding therapeutic decision-making [6]. Similarly, contemporary clinical reviews describe fibroid number, uterine volume, and submucosal or intramural location as consistent predictors of severe bleeding and an elevated likelihood of surgery [7].

Growing integration of artificial intelligence (AI) into clinical workflows allows decision support systems to standardize surgical decision-making through reproducible algorithmic reasoning [8]. Such systems are most valuable when they achieve high predictive accuracy while relying on a minimal number of easily obtainable clinical parameters—features that enhance practicality and integration into daily practice.

Previous efforts to develop machine learning (ML)-based UF decision tools have been limited by data quality and modest predictive performance. For example, Vetrivel et al. [9] trained models using publicly available Kaggle datasets and reported a maximum accuracy of 78%, underscoring the need for more clinically grounded, high-resolution datasets and externally validated models.

Recent studies have begun to explore the use of artificial intelligence-based decision support systems in uterine fibroid management. For example, an AI-driven clinical decision framework integrating female sex hormone parameters and fibroid characteristics has demonstrated promising results in predicting surgical necessity, achieving high concordance with expert clinical assessments [10]. These findings highlight the potential of computational models to translate complex clinical patterns into reproducible decision-support tools. However, most existing approaches rely on hormonal or laboratory parameters that may not always be routinely available in all clinical settings. Therefore, further investigation of simplified and widely accessible clinical predictors remains important for developing practical and scalable decision-support systems in everyday gynecologic practice.

The aim of this study was to develop an ML-based clinical decision helper algorithm to predict the need for myomectomy in patients with UFs using routinely available clinical, radiologic, and hematologic data, with particular emphasis on anemia-related parameters.

## 2. Methods

### 2.1. Ethical Consideration

This study was approved by the Istinye University Ethics Committee (30 June 2025; decision no. 24-18) and conducted in accordance with institutional regulations and the Declaration of Helsinki.

### 2.2. Patients

This study enrolled 618 patients diagnosed with UFs who were evaluated in the gynecology and obstetrics departments of three hospitals in Turkey. Of these patients, 238 underwent myomectomy. All analyses were planned to compare outcomes between the surgery and non-surgery cohorts. Because this study was designed as a retrospective multicenter analysis, the sample size was determined by the total number of eligible cases available in the participating institutions during the study period rather than by a predefined sample size calculation.

### 2.3. Study Design

This investigation was designed as a national, multicenter, retrospective study. A broad set of clinical and laboratory variables potentially associated with UF was curated for analysis. Hemoglobin (HGB) and ferritin concentrations were included as primary indicators of anemia, while additional inputs were incorporated into the machine learning workflow. Comprehensive statistical analyses and ML model training were performed to identify differences between the cohorts. Following the retrospective model development phase, a clinical concordance assessment procedure was performed.

The selection of clinical and biochemical variables included in the analysis was guided by clinical relevance, availability within the hospital information systems of the participating centers, and prior literature on determinants of surgical management in uterine fibroids. Variables related to fibroid burden (e.g., fibroid number and volume) and anemia status (hemoglobin and ferritin) were considered primary candidate predictors due to their established association with symptomatic severity and surgical intervention. Additional demographic and clinical parameters available in the dataset were also evaluated during the exploratory statistical phase to characterize the study population and support model development.

Uterine and fibroid volumes were estimated using the standard ellipsoid formula commonly applied in gynecologic imaging: volume = length × width × height × 0.523. These measurements were derived from routine imaging records available in the hospital databases.

Surgical intervention was performed according to routine clinical practice in the participating institutions. Indications for surgery generally included significant abnormal uterine bleeding associated with anemia, symptomatic large or multiple fibroids, mass-related symptoms, failure of conservative treatment, or patient preference following clinical evaluation. Final treatment decisions were made by the attending gynecologists based on standard clinical assessment.

### 2.4. Clinical Concordance Assessment with a Blinded Gynecologist

To assess the external performance of the trained ML model, an independent clinical concordance assessment was performed using 50 anonymized cases. A blinded gynecologist reviewed the dataset used for the clinical concordance assessment without access to the model predictions or the actual surgical decisions. Each case was assessed exclusively using available clinical variables—including UF number, UF volume, anemia-related parameters, gravidity, parity, and disease duration—to determine the indication for myomectomy.

### 2.5. Statistical Analyses and Machine Learning Training Tools

Statistical analyses and ML model training were conducted using Wistats v3.0 (WisdomEra Corp, İstanbul, Türkiye.), which is built on Python v2.7.14-based libraries. Data distribution was evaluated using skewness, kurtosis, and the Shapiro–Wilk test, and statistical methods were chosen accordingly.

Chi-square and Fisher’s exact tests were used to compare categorical variables. Numerical variables were analyzed via Kruskal–Wallis, independent-samples t-test, one-way ANOVA or Mann–Whitney U test based on distributional assumptions. Correlations were examined using Pearson or Spearman coefficients.

Predictive accuracy of the model was evaluated using multivariate logistic regression and additional ML algorithms integrated into the analytical workflow. A two-tailed *p*-value < 0.05 was considered statistically significant.

### 2.6. Machine Learning Workflow and Pipeline

Prior to ML model development, all statistical analyses were completed to characterize the dataset and guide variable selection. The primary outcome was myomectomy intervention status, which served as the basis for constructing algorithms to predict the need for surgery using clinical and laboratory data. Multiple ML models—including support vector machines (SVM), logistic regression, random forest, and decision tree—were trained and compared within the analytical framework.

**Figure 1:**
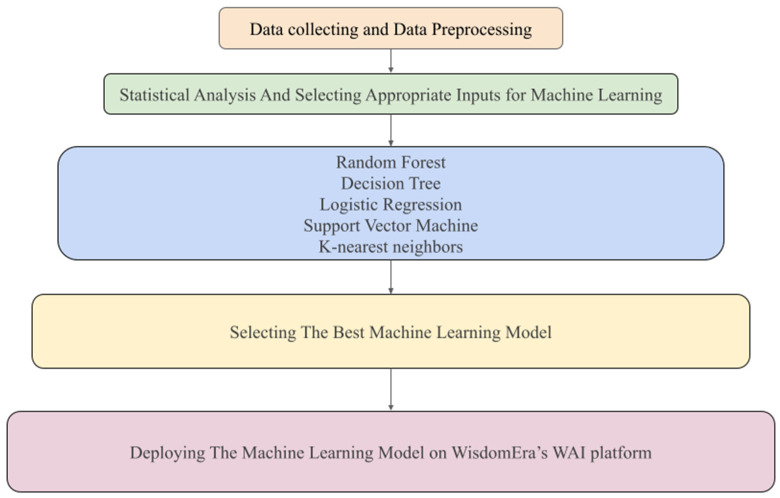
Machine learning workflow for predicting surgical necessity in women with uterine fibroids. A schematic representation of the analytical pipeline used in the study. The figure provides a structured overview of the methodological steps leading from raw data to model implementation.

A 70:30 split was used for training and test sets, respectively. ML model performance was evaluated using a comprehensive set of metrics, including accuracy, sensitivity, area under the receiver operating characteristic curve (AUC), precision, and F1 score. These indicators collectively informed the comparative assessment of predictive strength across models (Figure 1). In our study, we achieved significant accuracy rates especially with the support vector machine. SVM is a powerful method for building a classifier [11].

### 2.7. Overfitting Assessment

To ensure that the high predictive performance of the models did not reflect overfitting, we conducted a multi-layered evaluation of model generalizability. First, training and testing accuracies were compared for each model to assess internal consistency; closely aligned values were interpreted as evidence against overfitting. Second, five-fold cross-validation was applied to the training dataset to provide a more robust estimation of out-of-sample performance. The concordance between cross-validation accuracy, test accuracy, and the area under the ROC curve confirmed that the observed performance was stable across data partitions rather than driven by idiosyncratic patterns in a single split. Taken together, these analyses demonstrated that the models exhibited strong generalization capacity and that the high accuracy values were not attributable to overfitting.

### 2.8. Deploying the Best Decision Support Algorithm on the Application

**Figure 2:**
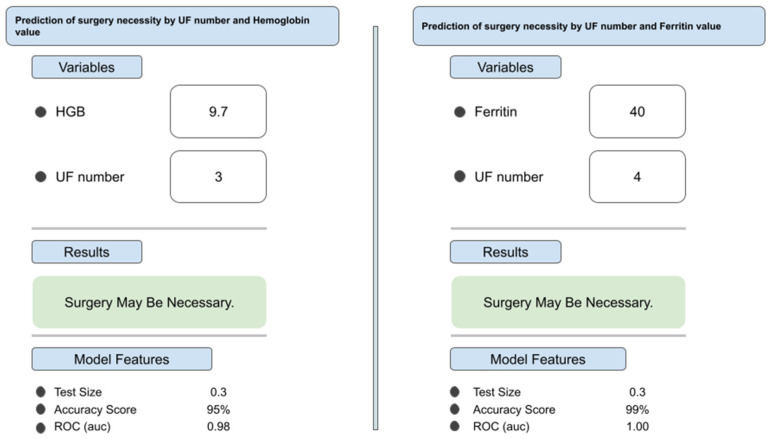
Example interface outputs demonstrating model predictions in two representative patient scenarios. Two independent sample cases illustrate how clinical inputs (hemoglobin, ferritin, myoma count, and myoma volume) generate individualized predictions regarding the necessity for surgical intervention.

Among the developed algorithms, it was decided that the machine learning model with the highest power could be used clinically, and the algorithm installation was carried out on the WisdomEra-WAI data analytics web template platform, which is on the cloud infrastructure (www.wisdomera.io) (accessed on 1 January 2026). In this application, we introduce many decision support algorithms that we have worked on and will link them to our articles. In addition, surgical necessity output can be obtained from the algorithms with input parameters. (Figure 2) Our algorithms can be integrated into decision support systems on a service basis through this application. Thus, we can offer them to the use of the specialists and patients (https://jinekoai.com) (accessed on 1 January 2026).

## 3. Results

### 3.1. Statistical Analysis Results

A comprehensive overview of descriptive and comparative statistical analyses is provided in Table 1. The surgery and non-surgery cohorts showed similar age distributions (35.7 vs. 35.4 years; *p* = 0.613). Similarly, disease duration did not differ significantly between cohorts (*p* = 0.361); the proportion of patients with symptoms lasting more than five years was 47% in the surgery group and 43% in the non-surgery group.

Anemia-related parameters demonstrated marked differences between the two groups. Mean hemoglobin levels were significantly lower in patients who underwent surgical treatment than in those managed conservatively (9.6 vs. 11.4 g/dL; *p* < 0.001). Ferritin levels showed a similar pattern, with surgically treated patients exhibiting substantially lower values (16.5 ng/mL vs. 66.7 ng/mL; *p* < 0.001).

Analysis of fibroid characteristics also revealed significant group differences. UF volume was significantly greater in the surgery cohort than in the non-surgery cohort (90.8 vs. 73.1 cm^3^; *p* < 0.001). In contrast, the number of fibroids did not differ significantly between groups (4.7 vs. 4.6; *p* = 0.384).

### 3.2. Machine Learning Results

A series of supervised ML models were developed to predict the necessity of surgical intervention in patients with UFs using multiple combinations of fibroid-related and anemia-associated parameters. Four feature sets were evaluated, and model performance was quantified using training accuracy, test accuracy, five-fold cross-validation accuracy, ROC–AUC, precision, sensitivity, and F1-score. The detailed metrics for each model are provided in Table 2.

**Figure 3:**
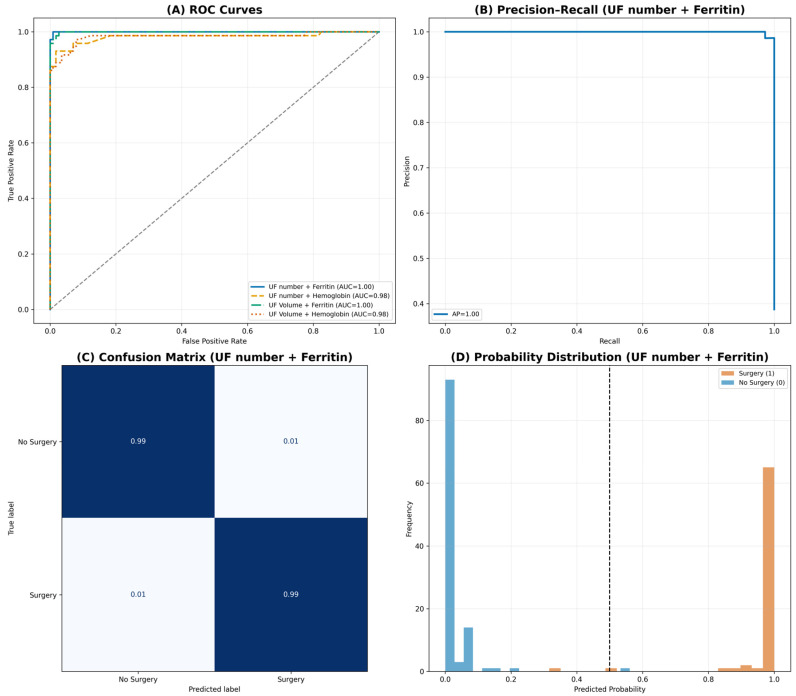
Comprehensive diagnostic performance of the best-performing support vector machine (SVM) model (‘Uterine Fibroid (UF) number + Ferritin’). The composite figure summarizes key performance characteristics of the highest-performing model. (**A**) ROC curves comparing all four feature combinations, demonstrating superior discrimination for ferritin-based models. (**B**) Precision–recall curve for the best-performing model, showing an average precision of 1.00. (**C**) Normalized confusion matrix indicating high classification accuracy with balanced performance across both classes. (**D**) Distribution of predicted probabilities stratified by true clinical outcome, illustrating clear separation around the decision threshold. Together, these visualizations demonstrate strong model discrimination without evidence of overfitting.

Across all configurations, the SVM models consistently emerged as the most effective classifier. Models incorporating ferritin demonstrated the highest overall predictive performance. Specifically, the “UF number + Ferritin’’ model achieved near-perfect discrimination, with a test accuracy of 0.99, cross-validation accuracy of 0.99, and an AUC of 1.00. Similarly, the “UF Volume + Ferritin’’ model yielded comparably strong performance, with a test accuracy of 0.98, cross-validation accuracy of 0.98, and an AUC of 1.00. These results highlight the substantial predictive contribution of iron-storage biomarkers in determining surgical necessity (Figure 3).

Models trained with hemoglobin as the secondary feature also performed well, though with comparatively lower sensitivity. The “UF number + Hemoglobin’’ and “UF Volume + Hemoglobin’’ models each demonstrated a test accuracy of 0.95 and cross-validation accuracies of 0.94 and 0.95, respectively, with AUC values of 0.98. Although precision remained high (0.98), sensitivity was moderately reduced (0.88), indicating a tendency of HGB-based models to under-detect certain surgical cases relative to ferritin-based models.

To ensure that the high accuracy values reported were not attributable to overfitting, we conducted a structured generalizability assessment. For each model, training accuracy was compared with test accuracy, followed by five-fold cross-validation performed on the training subset. The close agreement between train, test, and cross-validation accuracy across all models confirmed that the predictive performance was stable across multiple resampling conditions and did not reflect overfitting. The robustness of the ferritin-based models is further supported by their physiological relevance, given the well-established association between chronic iron-deficiency profiles and surgical indication in UF management.

**Figure 4:**
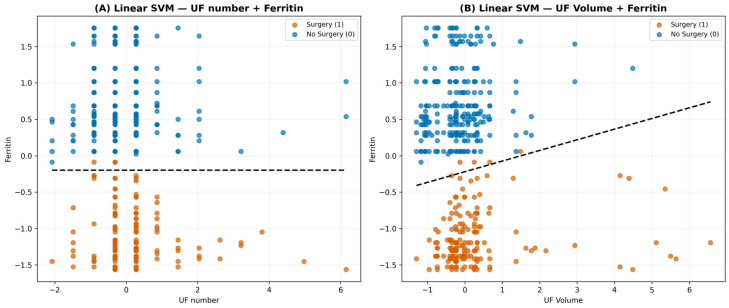
Linear Support Vector Machine (SVM) decision boundaries for two ferritin-based feature sets. Two panels showing linear decision boundaries using standardized feature spaces. (**A**) Uterine Fibroid (UF) number + Ferritin. (**B**) Uterine Fibroid (UF) volume + Ferritin. Each panel displays individual patient observations color-coded by true outcome, accompanied by the corresponding linear separating hyperplane. These plots visually demonstrate the contribution of ferritin to class separation and support the discriminative performance observed in nonlinear SVM analyses.

Importantly, to visualize the linear separability of the strongest feature combinations, we constructed two linear-kernel SVM decision-boundary plots (“UF number + Ferritin’’ and “UF Volume + Ferritin”). These plots illustrate that ferritin-based feature spaces produce clearer class margins with minimal overlap between surgery and non-surgery populations, providing an interpretable geometric representation of why these models outperform others. The linear SVM panels therefore offer an intuitive visual complement to the statistical performance metrics, reinforcing the discriminative strength of ferritin-integrated feature sets (Figure 4).

### 3.3. Clinical Concordance of Model Performance

**Figure 5:**
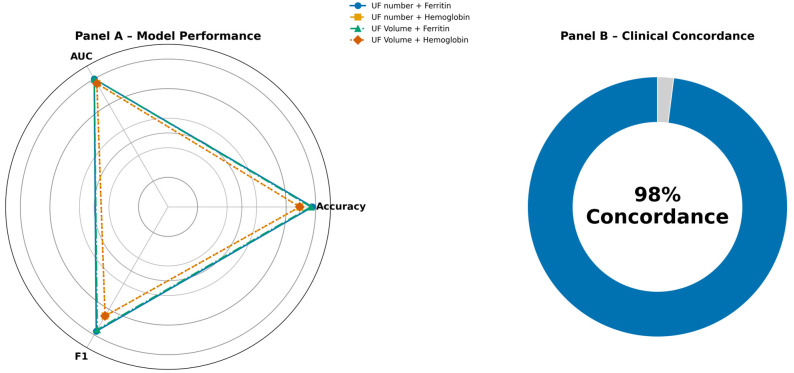
Comparing model performance and summarizing clinical concordance. (**A**) Radar chart comparing accuracy, AUC, and F1-score across the four feature combinations, indicating that ferritin-based models achieve the strongest and most balanced performance. (**B**) Donut plot illustrating the clinical concordance results, in which the model’s predictions aligned with the blinded gynecologist’s decisions in 49 out of 50 cases (98% concordance). This finding supports the model’s clinical applicability and generalizability. UF Number: Uterine Fibroid Number, UF Volume: Uterine Fibroid Volume.

Concordance was achieved in 49 of 50 cases, yielding an agreement rate of 98% (Figure 5). Further evaluation of the discordant case revealed a borderline clinical scenario with inherent uncertainty in surgical decision-making. The resulting high concordance indicates strong model robustness and close agreement with expert clinical assessment in real-world contexts.

## 4. Discussion

UFs have a substantial impact on women’s health, primarily through heavy menstrual bleeding and the development of iron-deficiency anemia [12]. These mechanisms contribute not only to symptomatic burden but also to progressive systemic effects that may necessitate surgical intervention. Timely surgical decision-making is therefore crucial; however, identifying the optimal timing for intervention remains challenging and requires an individualized approach. Single clinical indicators are often insufficient to reflect disease severity, particularly in patients with heterogeneous symptom profiles. Our findings align with established evidence showing that fibroid burden—particularly lesion number and volume—drives bleeding severity and hematologic decline. Structural parameters reflect mechanical and vascular effects of UFs, whereas hematologic indices capture the cumulative physiological impact over time. Hemoglobin, ferritin, and fibroid characteristics together formed a strong predictive basis for determining surgical necessity. This multidimensional assessment mirrors real-world clinical reasoning and supports more precise stratification of patients requiring operative management.

Across our ML models, combinations integrating UF volume or count with hematologic markers consistently produced high predictive accuracy, with ferritin-based models outperforming all other configurations. The very high discrimination metrics observed in the present analysis should therefore be interpreted cautiously, as they may partly reflect the strong clinical signal associated with severe anemia and fibroid burden rather than purely algorithmic performance. This finding suggests that iron storage depletion may represent a critical threshold marker rather than a secondary consequence of bleeding alone. These results underscore the physiological importance of iron-storage depletion as a crucial step linking symptomatic fibroids to operative necessity. The intentionally parsimonious structure of the models reflects an effort to balance predictive performance with interpretability and practical clinical applicability using universally available parameters. Beyond predictive performance, translating well-established clinical reasoning patterns into structured computational frameworks represents an important component of the digital transformation of healthcare. Even when certain clinical relationships are already recognized in routine practice, formalizing these patterns within reproducible algorithmic systems may contribute to the standardization of clinical reasoning, reduction in inter-clinician variability, and integration of established medical knowledge into digital decision-support infrastructures. Although surgical decision-making in uterine fibroids is inherently multifactorial, the present model intentionally focuses on a minimal set of highly informative predictors to maintain interpretability and facilitate potential integration into routine clinical workflows. Notably, the model’s performance extended beyond retrospective analysis, demonstrating stability when applied to real-time clinical decision scenarios. During prospective clinical concordance, the algorithm achieved 98% agreement with an independent, blinded gynecologist—49 of 50 cases—indicating that the system not only captures clinically meaningful patterns associated with disease severity but also closely reflects expert-level clinical reasoning. Such concordance supports the algorithm’s potential role as a reliable adjunct in complex decision-making environments. The high predictive performance observed in the present study may partly reflect the strong clinical signal associated with severe anemia and substantial fibroid burden. In such scenarios, surgical indications may already be clinically evident. Therefore, the proposed algorithm should be interpreted primarily as a framework for formalizing and standardizing clinically meaningful decision patterns rather than as a tool intended to replace established clinical judgment. Such alignment reinforces its potential utility in situations where subjective variability or limited clinical experience may hinder consistent decision-making. This comparison should therefore be interpreted as an assessment of clinical concordance rather than a strict methodological external validation. Because the blinded gynecologist had access to a broader set of clinical variables than those used by the machine learning models, the observed concordance should be interpreted as an exploratory comparison rather than as formal validation of model generalizability. In this context, algorithmic support may help standardize care without diminishing clinician autonomy. Nevertheless, the potential implications of model misclassification should be considered when interpreting algorithmic outputs. In clinical practice, a false-positive prediction (indicating surgery when conservative management may be appropriate) could theoretically lead to unnecessary surgical consideration, whereas a false-negative prediction (suggesting conservative management despite a surgical indication) might delay appropriate operative treatment. Importantly, the proposed algorithm is designed to function as a clinical decision-support tool rather than an autonomous decision-making system. Therefore, algorithmic predictions should always be interpreted together with clinical evaluation, imaging findings, and patient preferences. From an ethical perspective, the integration of AI into clinical decision-making requires careful consideration. Transparency of algorithmic reasoning, appropriate clinician oversight, and clear delineation of responsibility remain essential to ensure safe implementation. AI-based systems should therefore be viewed as supportive tools that assist clinicians in interpreting complex clinical patterns rather than as independent decision-makers. Responsible integration into clinical workflows requires maintaining physician accountability while leveraging the analytical strengths of data-driven models.

The increasing availability of ML and deep learning frameworks holds promise for addressing diagnostic uncertainty, personalizing treatment strategies, and improving outcomes. However, despite growing research interest, AI tools have yet to gain meaningful traction in daily gynecologic practice [13]. In this context, recent work has also explored AI-based clinical decision support approaches integrating hormonal parameters with fibroid characteristics. For instance, a multicenter study demonstrated that models combining female sex hormone levels with fibroid morphology could achieve high predictive performance and substantial clinical concordance with expert assessments when estimating surgical necessity in women with UFs [10]. These findings further support the concept that computational models can translate complex clinical patterns into reproducible decision-support frameworks. Barriers to adoption often include concerns regarding interpretability, workflow integration, and clinical trust. Prior studies highlight this potential. For instance, Hue et al. reported that an AI-assisted approach significantly enhanced the diagnostic accuracy of junior ultrasonographers for UFs, approaching the performance of senior specialists [14]. Similarly, other investigations have shown that AI systems can match expert-level accuracy when classifying benign versus malignant lesions [15] and can enhance the precision of hysteroscopic myomectomy by identifying the spatial localization of submucosal fibroids [16]. These applications predominantly focus on image-based diagnostics rather than therapeutic decision-making. Unlike prior AI studies focused primarily on imaging-based diagnosis or lesion classification, the present work is among the first to target surgical decision-making using routine clinical and hematologic parameters. By relying on commonly available data, the model enhances feasibility and supports broader clinical adoption.

Therapeutic decision-making in UF management is inherently multifactorial. As highlighted by Micić and colleagues, treatment typically begins with pharmacologic or minimally invasive approaches, although surgical intervention remains the most definitive and frequently applied option. These decisions require balancing risks and benefits while accounting for each patient’s symptoms, comorbidities, and individual preferences [5]. Such complexity often leads to inter-clinician variability, particularly in borderline cases. Our findings align with this framework: UF volume was significantly higher in the surgery cohort, underscoring its relevance as a determinant of operative need. This association reinforces the role of fibroid burden as a measurable driver of treatment escalation. This observation reinforces that fibroid burden continues to be a central driver of treatment escalation, regardless of interpatient variability. The decision support algorithm developed in this study may therefore aid clinicians—particularly those early in their careers—in navigating these complex choices with greater consistency and objectivity. Importantly, the goal of the proposed system is not to replace obvious clinical reasoning but to help standardize decision-making in borderline clinical situations where multiple moderate predictors interact and clinician experience may influence management strategies. The algorithm is therefore not intended to guide decisions in extreme scenarios where surgical indications are already clinically evident; rather, its potential value lies in supporting more consistent evaluation in cases where the indication for surgery may be less clear. By embedding established clinical determinants into a structured model, the algorithm supports reproducible and transparent decision-making. In this regard, the potential utility of such models may be particularly relevant in borderline clinical scenarios where the indication for surgery is not immediately evident and where clinician experience may influence management decisions.

The study has certain limitations. The etiology of anemia was assumed to be UF-related in most patients, although confirmatory postoperative hemoglobin or ferritin data were unavailable. This assumption reflects real-world clinical practice but limits etiologic specificity. Additionally, detailed information on preoperative medical or interventional treatments was not captured, as such data were absent from the database. The lack of treatment history restricts assessment of potential modifying effects on hematologic status. While these gaps limit the granularity of etiologic assessment, the overall pattern—markedly lower hematologic values in the surgery cohort—strongly suggests UF-related anemia as the predominant driver. Another limitation relates to the availability of detailed fibroid characteristics and symptom-level clinical data. Due to the retrospective multicenter design and differences in documentation across participating institutions, variables such as fibroid localization and detailed symptom profiles were not consistently available in the dataset and therefore could not be incorporated into the analyses. In addition, detailed information on concomitant gynecological conditions (such as endometriosis, adenomyosis, or endometrial hyperplasia) and systemic comorbidities (including hypertension, diabetes mellitus, or other metabolic disorders) was not consistently available across all centers and therefore could not be incorporated into the analyses. The consistency of this finding across analyses supports its clinical plausibility. Future studies incorporating longitudinal hematologic monitoring and detailed pretreatment histories could provide a more comprehensive understanding of causal mechanisms. Despite these limitations, the predictive performance and clinical concordance support the applicability of the proposed algorithm. Thus, the findings remain robust within the context of available data. Future investigations incorporating larger prospective cohorts and more granular clinical variables may allow specific evaluation of model behavior within intermediate-risk or “gray zone’’ decision spaces, where clinical judgment often varies between practitioners.

## 5. Conclusions

In conclusion, although the optimal timing of surgical intervention for uterine fibroids continues to vary across clinical settings, our findings suggest that key clinical determinants of surgical decision-making can be captured within a transparent algorithmic framework. By integrating fibroid burden with anemia-related biomarkers, the proposed model demonstrated strong discriminatory performance and a high level of clinical concordance with the assessment of an independent gynecologist.

Importantly, the algorithm is not intended to replace clinical judgment or guide decisions in obvious clinical scenarios. Rather, its potential value lies in supporting more consistent evaluation in situations where surgical indications may be less clear. In this context, AI-based tools may help reduce subjective variability and provide an additional structured perspective in the clinical assessment process.

While the current model represents a parsimonious and interpretable decision-support approach based on routinely available parameters, further prospective studies incorporating broader clinical variables and real-world implementation frameworks will be necessary to fully determine its impact on clinical decision-making and patient outcomes.

## Figures and Tables

**Table 1:** Baseline case characteristics and comparative clinical findings between the surgery and non-surgery groups.

	Surgery n (%)	No-Surgery n (%)	*p*
n	238 (38.5)	380 (61.5)	
Age (mean)	35.7	35.4	0.613
Hemoglobin (g/dL)	9.6	11.4	<0.001
Ferritin (ng/mL)	16.5	66.7	<0.001
UF number	4.7	4.6	0.384
UF volume (cc)	90.8	73.1	<0.001
Uterus volume (cc)	91.9	75.1	<0.001
Body Mass Index	29.1	29.0	0.686
Pregnancy Desire			
Yes	176 (74)	208 (55)	<0.001
No	62 (26)	172 (45)	
Gravidity			
Yes	212 (89)	327 (86)	0.322
No	26 (11)	53 (14)	
Parity			
Yes	148 (62)	267 (70)	0.043
No	90 (38)	113 (30)	
Disease Duration			
1–5 years	126 (53)	216 (57)	0.361
>5 years	112 (47)	164 (43)	

UF: Uterine Fibroid. n: Number of Cases.

**Table 2:** The Most Efficient Machine Learning Models.

Design	Model	Results
Inputs: UF number, Ferritin Output: Need for Surgery	Support Vector Machine	Train Accuracy: 0.99 Test Accuracy: 0.99 CV Accuracy: 0.99 ROC (AUC): 1.00 Precision: 1.00 Sensitivity: 0.97 F1 score: 0.99
Inputs: UF number, HGB Output: Need for Surgery	Support Vector Machine	Train Accuracy: 0.94 Test Accuracy: 0.95 CV Accuracy: 0.94 ROC (AUC): 0.98 Precision: 0.98 Sensitivity: 0.88 F1 score: 0.93
Inputs: UF Volume, Ferritin Output: Need for Surgery	Support Vector Machine	Train Accuracy: 0.99 Test Accuracy: 0.98 CV Accuracy: 0.98 ROC (AUC): 1.00 Precision: 0.97 Sensitivity: 0.99 F1 score: 0.98
Inputs: UF Volume, HGB Output: Need for Surgery	Support Vector Machine	Train Accuracy: 0.95 Test Accuracy: 0.95 CV Accuracy: 0.95 ROC (AUC): 0.98 Precision: 0.98 Sensitivity: 0.88 F1 score: 0.93

UF: Uterine Fibroid.

## Data Availability

The data supporting the findings of this study are hosted on the Istinye University Data Sharing Platform. Access to the fully de-identified dataset is provided via the platform https://dataset.istinye.edu.tr/dataset?did=60 (accessed on 1 January 2026) in compliance with applicable ethical standards and institutional data-use licensing requirements.

## References

[1] Yang Q., Ciebiera M., Bariani M.V., Ali M., Elkafas H., Boyer T.G., Al-Hendy A. (2022). Comprehensive Review of Uterine Fibroids: Developmental Origin, Pathogenesis, and Treatment. Endocr. Rev..

[2] Giuliani E., As-Sanie S., Marsh E.E. (2020). Epidemiology and management of uterine fibroids. Int. J. Gynecol. Obstet..

[3] Tinelli A., Morciano A., Sparic R., Hatirnaz S., Malgieri L.E., Malvasi A., D’amato A., Baldini G.M., Pecorella G. (2025). Artificial Intelligence and Uterine Fibroids: A Useful Combination for Diagnosis and Treatment. J. Clin. Med..

[4] Ahmad A., Kumar M., Bhoi N.R., Badruddeen, Akhtar J., Khan M.I., Ajmal M., Ahmad M. (2023). Diagnosis and management of uterine fibroids: Current trends and future strategies. J. Basic Clin. Physiol. Pharmacol..

[5] Micić J., Macura M., Andjić M., Ivanović K., Dotlić J., Micić D.D., Arsenijević V., Stojnić J., Bila J., Babić S. (2024). Currently Available Treatment Modalities for Uterine Fibroids. Medicina.

[6] Antunes D., Gante I., Carvalho M.J., Medeiros-Borges C., Águas F. (2022). The impact of anemia on treatment management and clinical outcomes of women hospitalized for uterine leiomyomas. Ginekol. Pol..

[7] Behairy M.S., Goldsmith D., Schultz C., Morrison J.J., Jahangiri Y. (2024). Uterine fibroids: A narrative review of epidemiology and management, with a focus on uterine artery embolization. Gynecol. Pelvic Med..

[8] Mills S. (2019). Electronic Health Records and Use of Clinical Decision Support. Crit. Care Nurs. Clin. N. Am..

[9] Vetrivel S., Rexline D., Gowri M.s.T.D. (2022). Decision Support Tool for Uterine Fibroids Treatment with Machine Learning Algorithms—A Study. Int. J. Sci. Res. Publ. IJSRP.

[10] Öz I., Yegin E.E., Öz A.U., Ulukaya E. (2026). An AI-Driven Clinical Decision Support Framework Utilizing Female Sex Hormone Parameters for Surgical Decision Guidance in Uterine Fibroid Management. Medicina.

[11] Huang S., Cai N., Pacheco P.P., Narandes S., Wang Y., Xu W. (2017). Applications of Support Vector Machine (SVM) Learning in Cancer Genomics. Cancer Genom. Proteom..

[12] Vannuccini S., Petraglia F., Carmona F., Calaf J., Chapron C. (2024). The modern management of uterine fibroids-related abnormal uterine bleeding. Fertil. Steril..

[13] Polat G., Arslan H.K. (2024). Artificial Intelligence in Clinical and Surgical Gynecology. İstanbul Gelişim Üniversitesi Sağlık Bilim. Derg..

[14] Huo T., Li L., Chen X., Wang Z., Zhang X., Liu S., Huang J., Zhang J., Yang Q., Wu W. (2023). Artificial intelligence-aided method to detect uterine fibroids in ultrasound images: A retrospective study. Sci. Rep..

[15] Wright D.E., Gregory A.V., Anaam D., Yadollahi S., Ramanathan S., Oyemade K.A., Alsibai R., Holmes H., Gottlich H., Browne C.-A.G. (2023). Developing a Machine Learning-Based Clinical Decision Support Tool for Uterine Tumor Imaging. arXiv.

[16] Chen M., Kong W., Li B., Tian Z., Yin C., Zhang M., Pan H., Bai W. (2023). Revolutionizing hysteroscopy outcomes: AI-powered uterine myoma diagnosis algorithm shortens operation time and reduces blood loss. Front. Oncol..

